# Supplementary material for: Comparative multiomics analysis of cell physiological state after culture in a basket bioreactor
Source: Sci Rep. 2022 Nov 23;12:20161. doi: 10.1038/s41598-022-24687-4 (PMC9686226; doi:10.1038/s41598-022-24687-4)

66048.1:123468331:123468597:-@NW_023666048.1:123468331:123468614:-@NW_023666048.1:123471769:123

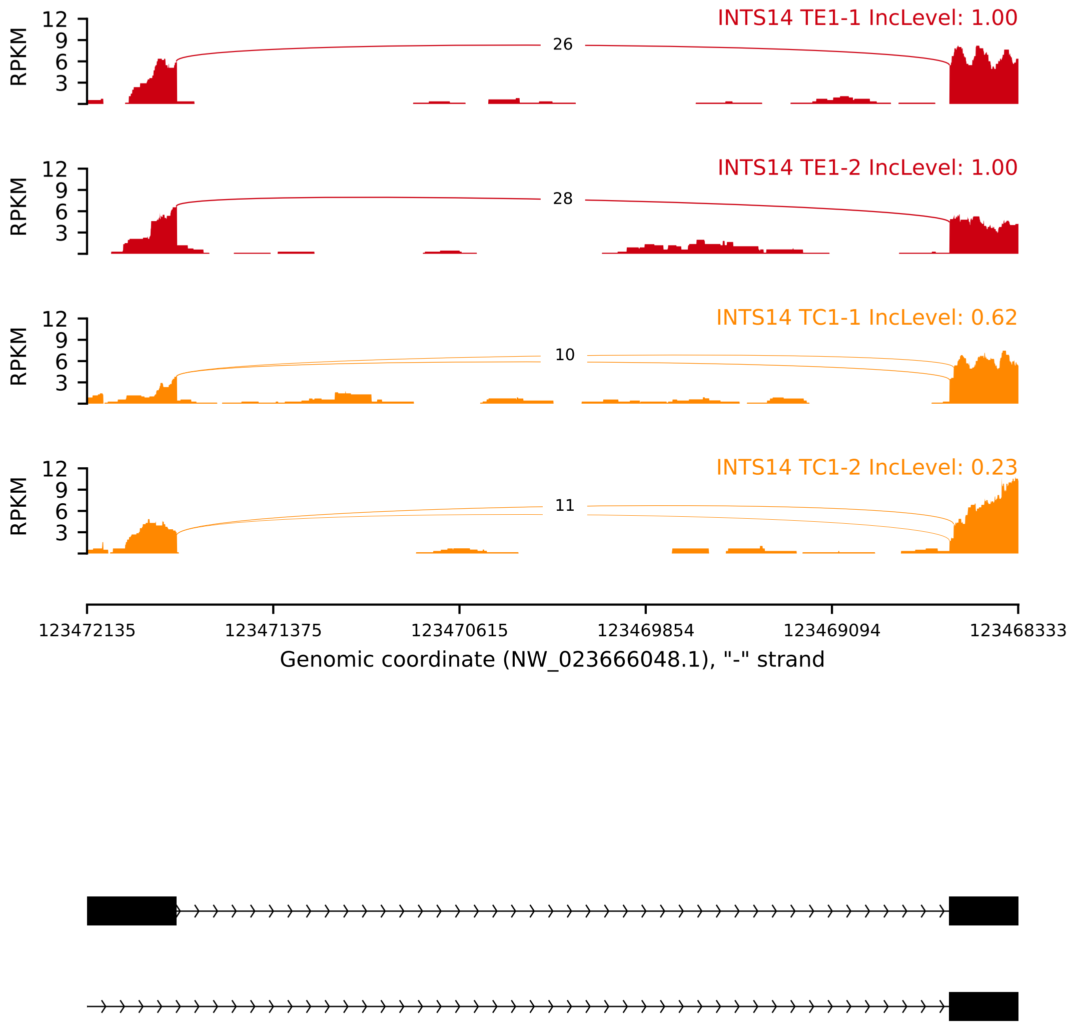

Supplement: Supplementary file 1 — Supplementary Information 1. [file 41598_2022_24687_MOESM1_ESM.zip › raw data/Transcriptomic raw data/7.AS/2.dasplot/TE1vsTC1/A3SS/16_INTS14_NW_023666048.1_123468331_123468597_-@NW_023666048.1_123468331_123468614_-@NW_023666048.1_123471769_123472134_-.pdf]
